# Mitochondrial Redox Status Regulates Glycogen Metabolism via Glycogen Phosphorylase Activity

**DOI:** 10.3390/antiox13111421

**Published:** 2024-11-20

**Authors:** Ikko Sakamoto, Shuichi Shibuya, Hidetoshi Nojiri, Kotaro Takeno, Hiroshi Nishimune, Keisuke Yaku, Takashi Nakagawa, Muneaki Ishijima, Takahiko Shimizu

**Affiliations:** 1 Department of Medicine for Orthopaedics and Motor Organ, Juntendo University Graduate School of Medicine, Tokyo 113-0034, Japan; 16640sakamoto@sbc.or.jp (I.S.); hnojiri@juntendo.ac.jp (H.N.); ishijima@juntendo.ac.jp (M.I.); 2 Aging Stress Response Research Project Team, National Center for Geriatrics and Gerontology, Aichi 474-8511, Japan; s-shibuya@rs.socu.ac.jp; 3 Department of Regenerative Medicine, Faculty of Pharmacy, Sanyo-Onoda City University, Yamaguchi 756-0884, Japan; 4 Laboratory of Neurobiology of Aging, Tokyo Metropolitan Institute for Geriatrics and Gerontology, Tokyo 173-0015, Japan; takeno@tmig.or.jp (K.T.); nishimun@tmig.or.jp (H.N.); 5 Department of Applied Biological Science, Tokyo University of Agriculture and Technology, Tokyo 183-8538, Japan; 6 Department of Molecular and Medical Pharmacology, Faculty of Medicine, University of Toyama, Toyama 930-8555, Japan; yaku@med.u-toyama.ac.jp (K.Y.); nakagawa@med.u-toyama.ac.jp (T.N.); 7 Department of Food and Reproductive Function Advanced Research, Juntendo University Graduate School of Medicine, Tokyo 113-8421, Japan

**Keywords:** mitochondria, SOD2, redox balance, glycogen, glycogen phosphorylase

## Abstract

Mitochondria and glycogen are co-distributed in skeletal muscles to regulate the metabolic status. Mitochondria are also redox centers that regulate the muscle function during exercise. However, the pathophysiological relationship between the mitochondrial redox status and glycogen metabolism in the muscle remains unclear. In the present study, we examined the pathological effects of mitochondrial dysfunction induced by mitochondrial superoxide dismutase (SOD2) depletion on glycogen metabolism. We found that muscle glycogen was significantly accumulated in association with motor dysfunction in mice with a muscle-specific SOD2 deficiency. Muscle glycogen phosphorylase (GP-M) activity, which is a key enzyme for glycogen degradation at times when energy is needed (e.g., during exercise), was significantly decreased in the mutant muscle. Moreover, the GP-M activity on normal muscle sections decreased after treatment with paraquat, a superoxide generator. In contrast, treatment with antioxidants reversed the GP-M activity and motor disturbance of the mutant mice, indicating that GP-M activity was reversibly regulated by the redox balance. These results demonstrate that the maintenance of the mitochondrial redox balance regulates glycogen metabolism via GP-M activity.

## 1. Introduction

Skeletal muscles produce energy to promote muscle contraction, primarily through mitochondrial respiration and glycolysis. Mitochondrial respiration uses a proton concentration gradient in the electron transport chain to produce ATP. Glycolysis decomposes glucose into organic substances such as pyruvate to produce ATP. Aging leads to a decline in the muscle function, including muscle weakness and sarcopenia. The mitochondrial function and activity of glycogen phosphorylase (GP-M), the rate-limiting enzyme of glycogenolysis in muscles, decrease with aging [1], suggesting a strong correlation between muscle function and the energy production system.

Reactive oxygen species (ROS) can cause oxidative damage to tissues, resulting in various diseases. Aging rats have increased oxidative damage markers in skeletal muscles [2], indicating a correlation between muscle dysfunction and the accumulation of ROS. Mammals possess multiple antioxidant systems to prevent oxidative damage caused by ROS accumulation. Superoxide dismutase 2 (SOD2), an antioxidant enzyme, is constitutively and ubiquitously expressed in the mitochondria to regulate the redox balance in tissue cells. The loss of SOD2 induces mitochondrial redox imbalance by increasing superoxide generation, resulting in mitochondrial dysfunction in cells and several tissues, including the brain, heart, muscle, and bone [3,4,5]. In particular, muscle-specific SOD2-deficient (muscle-*Sod2*^−/−^) mice exhibit severe exercise intolerance and are used as a model of muscle fatigue [3,6]. These data indicate that the mitochondrial redox balance contributes to the mitochondrial and muscle functions in mice.

Muscle glycogen is one of the primary energy sources for glycolysis during exercise. Muscle fatigue is correlated with glycogen depletion [7,8]. McArdle disease, a glycogen storage disease, is characterized by a marked decline in exercise capacity due to abnormal glycogen metabolism caused by the decreased activity of muscle glycogen phosphorylase (GP-M), a limiting enzyme in glycogenolysis [9,10,11]. Muscle-*Sod2*^−/−^ mice exhibit severe exercise intolerance similar to McArdle disease [3]. However, the pathophysiological relationship between glycogen metabolism and the mitochondrial redox status in muscles is poorly understood.

In the present study, we examined the pathological effects of the mitochondrial redox imbalance caused by mitochondrial SOD depletion on the glycogen metabolism in the skeletal muscle of genetically modified mice. We also investigated the rescue effect of pharmacological intervention on the glycogen metabolism in vivo and ex vivo and discussed the biological relationship between the mitochondrial redox and glycogen-mediated metabolism during physical activity.

## 2. Materials and Methods

### 2.1. Animals and Genotyping

The generation of muscle-*Sod2*^−/−^ mice has been described previously [3]. Briefly, the neomycin resistance gene and exon 3 were flanked by loxP and deleted from the genome using Cre recombinase. The crossbreeding of homozygous Mn-SOD^lox/lox^ mice with HSA-Cre transgenic mice resulted in muscle-*Sod2*^−/−^ mice. Muscle-*Sod2*^−/−^ mice exhibited increased ROS accumulation in skeletal muscle, reduced mitochondrial respiration in muscle fibers, exercise intolerance, and reduced muscle regenerative capacity (Figure S1A,B). Male animals (age: 5–6 months) were maintained under a 12 h light/12 h dark cycle with ad libitum access to water and chow. The genotyping of the HSA-Cre transgene and muscle-*Sod2*^−/−^ mice was performed by PCR, using genomic DNA isolated from the tail tip [3]. Mice were maintained and studied according to the protocols approved by the National Center for Geriatrics and Gerontology.

### 2.2. Frozen Sections

Methyl butane was sufficiently cooled with dry ice containing hexane to freeze the tissue. The frozen tissue was sealed in an O.C.T. compound (Sakura Finetek Japan Co., Ltd., Tokyo, Japan) to make a block. Frozen sections were prepared at a thickness of 6 µm using a cryostat.

### 2.3. Glycogen Phosphorylase Activity Assay (GP-M Activity)

GP-M activity staining was performed as previously described [12]. We selected the gastrocnemius muscle for the analysis, which showed a marked accumulation of ROS, an increase in the central nucleus of muscle regeneration markers, and a decrease in mitochondrial respiratory capacity in muscle-*Sod2*^−/−^ mice [3]. Gastrocnemius samples were sealed in an O.C.T. compound in cold methylbutane. GP-M activity staining was performed only in the gastrocnemius muscle. For staining, muscle sections (6 µm) were incubated for 45 min in a solution containing 1% glucose-1-phosphate (G1P, Kanto Chemical Co., Inc., Tokyo, Japan), 0.2% AMP (Tokyo Chemical Industry Corporation, Tokyo, Japan), and 0.02% glycogen (Nacalai Tesque, Tokyo, Japan) in 0.1 M sodium acetate buffer (pH 5.6). The sections were washed with water, and Lugol’s iodine (Nacalai Tesque) was applied for 3 min to detect the glycogen bound to GP-M. These skeletal muscle sections were observed in bright-field using a fluorescent microscope (BZ-X; Keyence, Osaka, Japan). The staining density was quantified using an imaging analysis software program (Leica Q Win, Wetzlar, Germany). GP-M activity of muscle lysates was quantified by measuring the amount of G1P produced using a G1P assay kit (K697-100; BioVision # K697-100, San Francisco, CA, USA).

### 2.4. Periodic Acid–Schiff (PAS) Staining

Glycogen content was analyzed with PAS staining. Briefly, frozen sections (6 µm) were dried and immersed in a 0.5% periodic acid solution (FUJIFILM Wako Pure Chemical, Neuss, Germany) at room temperature for 7 min. After washing 5 times with purified water, they were immersed in Schiff reagent (Nacalai Tesque) and incubated at 37 °C for 15 min.

### 2.5. Glycogen Content

The glycogen content of the mouse gastrocnemius muscle was determined using a glycogen assay kit (#700480; Cayman Chemical, Ann Arbor, MI, USA).

### 2.6. Treatment with Vitamin B6

Vitamin B6 (VB6; Pyridoxine HCl; Nacalai Tesque) was dissolved in PBS at (a concentration of 10 mM). VB6 was administered either orally using a sonde or intraperitoneally using a 30 G needle. VB6 was continuously administered at a dose of (2 mg/kg) [13] for 5 days. The treadmill task was carried out following treatment on the 5th day.

### 2.7. Treadmill Protocol

A treadmill apparatus (MK-680S/OP; Muromachi Kikai, Tokyo, Japan) was used to determine the endurance capacity for running. An electrode was activated at the back of the treadmill to prevent mice from stopping naturally. The running test was performed at 12 m/min with a 0° slope. In our previous report, muscle-*Sod2*^−/−^ mice showed significant fatigue within 10 min of initiating the treadmill protocol [3,6]. The time until the mice could no longer stand up or move forward was measured as time-to-exhaustion. All mice were trained twice a few days before the actual performance test. The training runs were performed using the protocol described above, and the mice were allowed to run until they were no longer able to run (approximately 5–15 min).

### 2.8. Western Blotting

Muscle samples were lysed in NP-40 lysis buffer (50 mM Tris-HCl, pH 8.0; 150 mM NaCl; 1% NP-40; containing a protease inhibitor cocktail (Roche Diagnostics, Tokyo, Japan) and phosphatase inhibitors (Roche). The supernatants were collected, and 10 µg of protein from each sample was loaded onto a 10% SDS-polyacrylamide gel. Antibodies against muscle–glycogen phosphorylase (GP-M (1:1000, #88078; Abcam, Cambridge, MA, USA), total glycogen synthase (GS (1:500, #3886; Cell Signaling Technology, Danvers, MA, USA), phospho GS (p-GS; Ser 641, 1:500, #3891; Cell Signaling Technology), and GAPDH (1:1000, #2118S; Cell Signaling Technology) were used.

### 2.9. Quantitative Real-Time PCR

Total RNA was extracted from the muscles using TRIzol reagent (Thermo Fisher Scientific, Waltham, MA, USA) according to the manufacturer’s instructions. cDNA was synthesized from 1 µg of total RNA using reverse transcriptase (ReverTra Ace qPCR RT Master MIX; TOYOBO, Osaka, Japan). Real-time PCR was performed using a Mini Opticon (Bio-Rad, Hercules, CA, USA) with SYBR GREEN PCR Master Mix (Bio-Rad) according to the manufacturer’s instructions. All data were normalized to the level of the housekeeping gene, histone H2A. The primer sets used in this study are listed in Table 1.

### 2.10. Transfection and Treatment of Cells

HEK293 cells were transfected with the human *PYGM* (h*PYGM*) expression vector (#RC212365, OriGene Technologies, Rockville, MD, USA) using Lipofectamine 3000 (Thermo Fisher Scientific, Waltham, MA, USA), according to the manufacturer’s instructions. At 72 h after transfection, the cells were treated with 250 µM H_2_O_2_ for 1 h and then collected. The collected cells were lysed in assay buffer (1 mM AMP, 0.25% glycogen, 2 mM EDTA, 0.8 mM NADP+ [FUJIFILM Wako Pure Chemical], 10 mM magnesium acetate [FUJIFILM Wako Pure Chemical], 5 µM glucose 1,6-diphosphate [FUJIFILM Wako Pure Chemical], 5 units of glucose-6-phosphate dehydrogenase [FUJIFILM Wako Pure Chemical], and 5 units of phosphoglucomutase [FUJIFILM Wako Pure Chemical]). The cell lysate was incubated with 0.25% glycogen and 10 mM dithiothreitol (Tokyo Chemical Industry corporation) at 37 °C for 2 h. GP-M activity was performed using a G1P assay kit.

### 2.11. Treatment of Prooxidant and Antioxidants on Muscle Sections

Methyl viologen dichloride hydrate (paraquat; Merck, Darmstadt, Germany) was dissolved in PBS to create a 10 mM stock solution and was used at a final concentration of 100 µM for 5 min. EUK-134 (Axon MEDCHEM, Groningen, The Netherlands) was dissolved in PBS to create a 10 mM stock solution and was used at a final concentration of 50 µM for 45 min on muscle sections (6 µm). PAPLAL, a mixture of platinum and palladium nanoparticles with strong SOD and catalase activities [14], was provided by Toyokose Pharmaceutical Co. (Tokyo, Japan) and Musashino Pharmaceutical Co. (Tokyo, Japan). PAPLAL is composed of a mixture of 0.2 mg/mL (1.03 mM) nPt and 0.3 mg/mL (2.82 mM) nPd. PAPLAL was dissolved in PBS and used at final concentrations of 20 µg/mL nPt and 30 µg/mL nPd for 45 min on muscle sections.

### 2.12. Glucose Tolerance Test

Blood glucose concentration was measured using an automatic monitor (Glucocard; Arkray, Kyoto, Japan). During the glucose tolerance tests, mice were fasted for 24 h and then received an intraperitoneal injection of 20% D-glucose (2 g/kg body weight; Wako). Blood glucose concentrations were measured in whole blood obtained from the tail vein at 0, 15, 30, 60, and 120 min after glucose injection.

### 2.13. Immunohistochemistry and Imaging Analysis of Neuromuscular Junctions (NMJs)

The following antibodies were used: neurofilament (2H3, DSHB, Iowa City, IA, USA), SV2 (SV2, DSHB), Alexa Fluor 488 conjugated secondary antibody, and Alexa Fluor 594-conjugated α-bungarotoxin (Thermo Fisher Scientific). Immunohistochemical analyses were performed previously [15]. Briefly, the mice were fixed by transcardiac perfusion with 2% paraformaldehyde in PBS. Muscles were removed and post-fixed in 2% paraformaldehyde at room temperature, washed with PBS, and cryoprotected in 20% sucrose/PBS before being frozen in Optimal Cutting Temperature compound (Sakura, Torrance, CA, USA), and sections were cut using a cryostat (longitudinal for muscles). Muscles were sectioned at a thickness of 20 μm and blocked in PBS containing 2% bovine serum albumin (BSA), 2% normal goat serum, and 0.1% Triton X-100. The sections were then incubated with primary antibodies for 1 day at room temperature, washed with PBS, and incubated with appropriate secondary antibodies for 2 h at room temperature. The muscle sections were also incubated with Alexa Fluor 594-conjugated α-bungarotoxin. The sections were then washed with PBS and mounted using ProLong Glass Antifade Mountant (Thermo Fisher Scientific).

### 2.14. Analysis of NMJs

The innervation rate analysis of NMJs has been previously described [15]. Briefly, muscle sections were stained with antibodies against motor nerves (anti-neurofilament and anti-SV2) and Alexa Fluor 594-conjugated α-bungarotoxin for acetylcholine receptors. Adult motor nerve terminals showed perfect overlap with acetylcholine receptor clusters, indicating fully innervated NMJs. NMJs were assessed for areas of the acetylcholine receptor clusters that were not occupied by nerves, whether in part or in full, as partially innervated NMJs or denervated NMJs. The quantifications were from three to five mice of each genotype, with an average of 60 NMJs per animal. The observer was blinded to the genotype.

### 2.15. Statistical Analyses

Statistical analyses were performed using Student’s *t*-test for comparisons between two groups and one-way analysis of variance and Tukey’s test for comparisons between trees or more groups. *p* values of <0.05 were considered to indicate statistical significance. All data are expressed as the mean ± standard deviation (SD).

## 3. Results

### 3.1. Muscle-Specific Sod2-Deficient Mice Showed Impaired Glycogen Metabolism Due to Reduced GP-M Activity in Muscle

**Figure 1:**
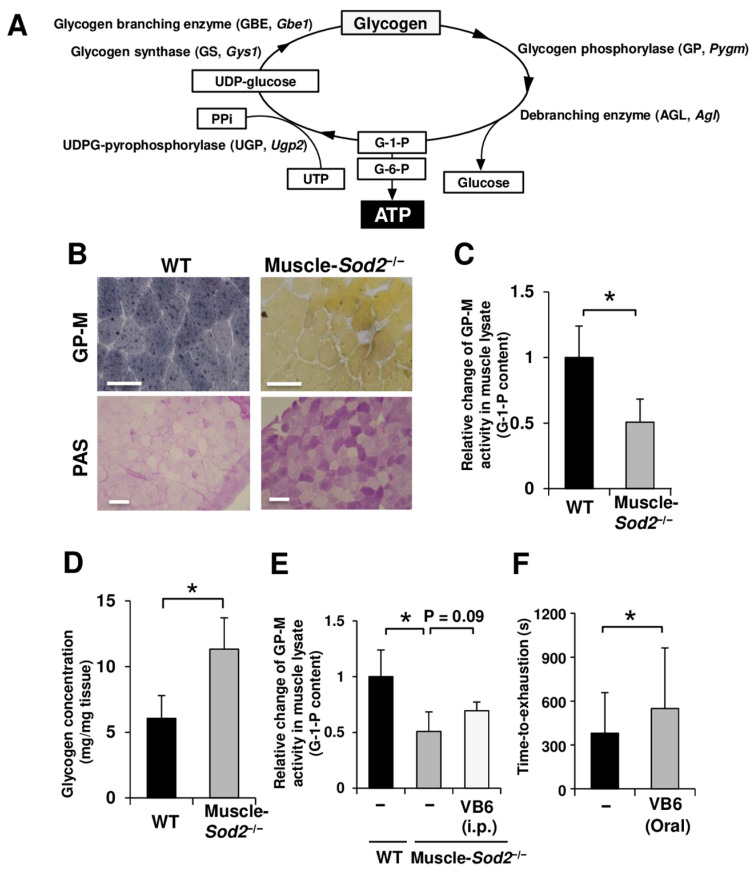
Muscle-*Sod2*^−/−^ mice, a muscle fatigue model, exhibit abnormal glycogen metabolism due to decreased GP-M activity. (**A**) A schematic illustration of the metabolic cycle of glycogen. (**B**) GP-M activity staining (upper) and periodic acid–Schiff (PAS) staining (under) of the gastrocnemius muscle of WT or muscle-*Sod2*^−/−^ mice. Scale bars represent 50 µm (GP-M) and 200 µm (PAS), respectively. (**C**) Biochemical quantification of GP-M activity of gastrocnemius muscle lysates of WT (*n* = 4) and muscle-*Sod2*^−/−^ (*n* = 4) mice. (**D**) The glycogen concentration in the gastrocnemius muscle of WT (*n* = 3) or muscle-*Sod2*^−/−^ mice (*n* = 3). (**E**) The biochemical quantification of the GP-M activity of muscle treated with or without VB6 (*n* = 4). VB6 was continuously administered by intraperitoneal injection at a dose of 2 mg/kg for 5 days. (**F**) The performance test of the muscle-*Sod2*^−/−^ (*n* = 10) and VB6-treated group (*n* = 10) at 5 months of age. VB6 was continuously administered by oral sonde for 5 days at a dose of 2 mg/kg. On the 5th day of treatment, the performance test was performed. Data indicate the mean ± SD; * *p* < 0.05.

Glycogen metabolism is a major source of energy supply in the skeletal muscle (Figure 1A). To investigate the effect of glycogen metabolism on the impaired motor function of muscle-*Sod2*^−/−^ mice, we examined GP-M activity of skeletal muscle using activity staining and biochemical assay. Activity experiments revealed that the GP-M activity in the *Sod2*^−/−^ muscles was markedly lower than that in the WT muscles (Figure 1B,C). Consistent with the decreased GP-M activity, the glycogen content in the muscles of mutant mice was significantly higher than that in comparison to WT mice (Figure 1B,D). VB6 is a coenzyme for GP-M and is also used as a treatment for McArdle disease [13,16,17]. The administration of VB6 to muscle-*Sod2*^−/−^ mice improved running activity, accompanied by an increasing trend of GP-M activity (Figure 1E,F), indicating that GP-M activity is a target for improving the motor function in muscle-*Sod2*^−/−^ mice.

**Figure 2:**
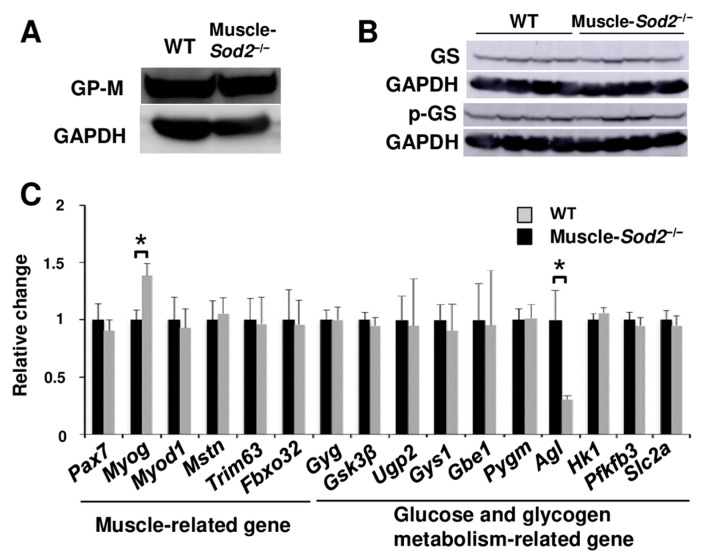
The expression of glycogen metabolic enzymes is not altered in the skeletal muscle of Muscle-*Sod2*^−/−^ mice. (**A**) Western blotting of GP-M protein in WT and muscle-*Sod2*^−/−^ mice. Protein extracts from the gastrocnemius muscle of WT and muscle-*Sod2*^−/−^ were immunoblotted with anti-GP-M and anti-GAPDH antibodies. (**B**) Western blotting of total GS (GS) and phospho-Ser641 GS (p-GS) in the gastrocnemius muscle. GAPDH was used as a loading control. (**C**) The relative mRNA levels of muscle-related genes (*Pax7*; paired box 7, *Myog*; myogenin, *Myod*; myogenic differentiation 1, *Mstn*; myostatin, *Trim63*; tripartite motif-containing 63, *Fbxo32*; atrogin-1), glucose and glycogen metabolism-related genes (*Gyg*; glycogenin, *Gsk3b*; glycogen synthase kinase 3 beta, *Ugp2*; UDP-glucose pyrophosphorylase 2, *Gys1*; glycogen synthase 1, muscle, *Gbe1*; glucan (1,4-alpha-), branching enzyme 1, *Pygm*; muscle glycogen phosphorylase, *Agl*; amylo-1,6-glucosidase, *Hk1*; hexokinase 1, *Pfkfb3*; 6-phosphofructo-2-kinase/fructose-2,6-biphosphatase 3, *Slc2a*; Glucose transporter 4) were measured by quantitative RT-PCR (*n* = 5). The data indicate the mean ± SD; * *p* < 0.05.

Next, we investigated the expression of the enzymes involved in glycogen metabolism. Western blotting indicated positive signals of the GP-M, total glycogen synthase (GS), and phospho-GS (p-GS; Ser 641, active form) protein in mutant mice (Figure 2A,B). Next, we estimated the expression levels of muscle-related and glucose and glycogen metabolism-related genes in the skeletal muscle. Consistent with the protein expression, the *Gys* and *Pygm* gene expression in WT and mutant mice did not differ to a statistically significant extent (Figure 2C). *Agl*, a glycogen debranching enzyme that acts downstream of GP-M, was significantly decreased in the muscles of mutant mice relative to those of WT mice (Figure 2C), suggesting a decrease in glycogenolysis due to reduced GP-M activity. *Myog* was also the only gene showing significantly increased expression levels, supporting the enhancement of muscle regeneration in mutant mice [3]. These results indicate that GP-M activity, but not the expression levels of GP-M and GS, was reduced in the skeletal muscle of mutant mice.

### 3.2. GP-M Activity Was Reversibly Regulated by Redox State

**Figure 3:**
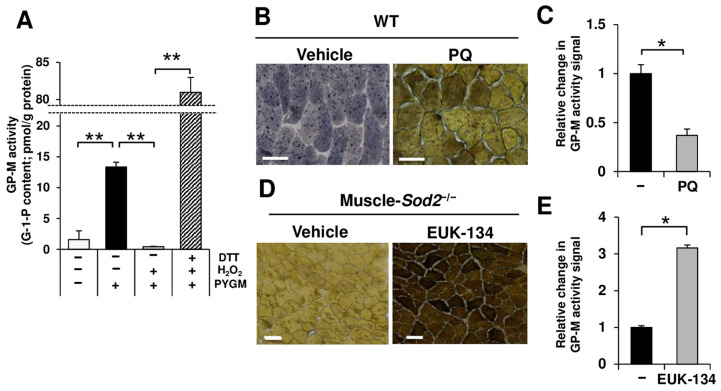
GP-M activity is reversibly regulated by the redox state. (**A**) GP-M activity of h*PYGM* overexpressed HEK293 cell lysate treated with or without H_2_O_2_ or DTT (*n* = 3). (**B**) GP-M activity staining of gastrocnemius muscle sections of WT mice treated with 100 µM paraquat for 5 min. Scale bars represent 50 µm. (**C**) The quantification of the staining intensity of (**B**) (*n* = 3). (**D**) GP-M activity staining of gastrocnemius muscle sections of muscle-*Sod2*^−/−^ mice treated with 50 µM EUK-134 for 45 min. Scale bars represent 50 µm. (**E**) The quantification of the staining intensity of (**D**) (*n* = 3). Data are shown as the mean ± SD; * *p* < 0.05, ** *p* < 0.01.

To examine the relationship between GP-M activity and the redox state, we measured GP-M activity by adding hydrogen peroxide to HEK293 cells overexpressing h*PYGM*. Cells overexpressed h*PYGM* showed significantly increased GP-M activity relative to control cells (Figure 3A). In contrast, treatment with hydrogen peroxide almost completely inactivated GP-M activity in cells overexpressing h*PYGM* (Figure 3A). Furthermore, the addition of DTT, a reducing agent, to the cell lysate collected after hydrogen peroxide treatment significantly restored GP-M activity (Figure 3A). suggesting that GP-M activity is constantly inactivated to some extent by oxidation. We further investigated whether ROS directly affects GP-M activity ex vivo. We performed the GP-M activity staining of frozen muscle sections of WT mice in the presence of paraquat, a superoxide generator. Importantly, paraquat-treated sections showed a significant decrease in staining intensity (Figure 3B,C). In contrast, mutant sections treated with EUK-134, an SOD mimetic, showed a significant increase in staining intensity (Figure 3D,E). We reconfirmed that a single injection of EUK-134 into mutant mice showed a markedly prolonged running time (Figure S2), demonstrating an improvement in their motor function.

**Figure 4:**
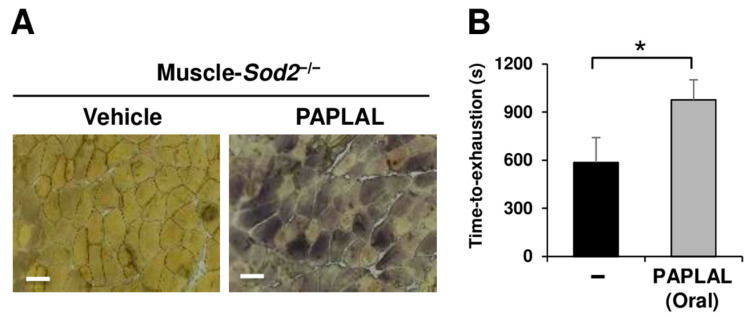
Antioxidant improves the motor function of muscle-*Sod2*^−/−^ mice by increasing GP-M activity. (**A**) GP-M activity staining of gastrocnemius muscle sections of muscle-*Sod2*^−/−^ mice treated with 1× PAPLAL for 45 min. Scale bars represent 200 µm. (**B**) The performance test was performed 1 h after the oral administration of 1× PAPLAL (10 mL/kg) to muscle-*Sod2*^−/−^ mice (*n* = 6). Data are shown as the mean ± SD; * *p* < 0.05.

We further investigated the effects of another antioxidant, PAPLAL [14], on GP-M activity. Treatment with PAPLAL increased the GP-M activity in sections of mutant muscle (Figure 4A). Consistent with GP-M activity, the oral administration of PAPLAL significantly increased the forced running ability of muscle-*Sod2*^−/−^ mice (Figure 4B). These results demonstrate that GP-M activity was impaired by oxidation and that it was reversibly rescued by antioxidants.

### 3.3. Muscle-Specific Sod2-Deficient Mice Showed Normal Glucose Tolerance and Innervation of the Neuromuscular Junction

**Figure 5:**
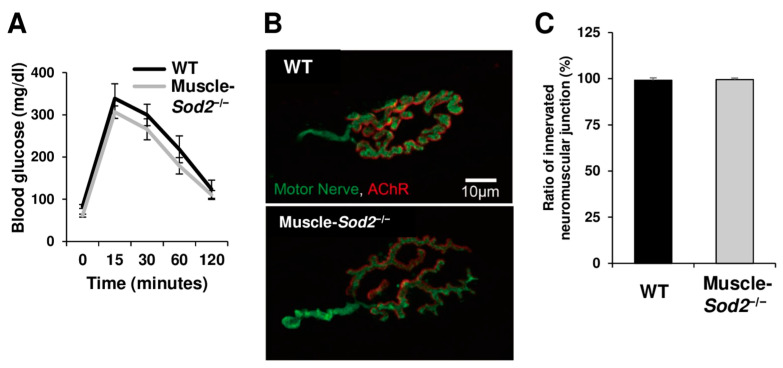
Muscle-*Sod2*^−/−^ mice show normal glucose tolerance and innervation of neuromuscular junctions. (**A**) Fasting blood glucose levels in WT (*n* = 5) and muscle-*Sod2*^−/−^ mice (*n* = 5), as determined by a glucose tolerance test at 5 months of age. (**B**) Immunohistochemical staining of neurofilament and SV2 (Motor Nerve), and acetylcholine receptor (AChR) in neuromuscular junctions (NMJs) of WT or muscle-*Sod2*^−/−^ mice. (**C**) The innervation rate analysis of 47–78 NMJs of WT (*n* = 5) or muscle-*Sod2*^−/−^ (*n* = 3) mice. Data are shown as the mean ± SD.

To clarify other mechanisms contributing to motor disturbances in muscle-*Sod2*^−/−^ mice, we next examined the glucose metabolism of mutant mice using a glucose tolerance test. The glucose tolerance of the control and mutant mice did not differ to a statistically significant extent (Figure 5A). We further examined the neuromuscular junctions by immunofluorescence staining. No differences were observed in the ratio of neuromuscular junctions between WT and muscle-*Sod2*^−/−^ mice, indicating innervation (Figure 5B,C). These results indicate that motor disturbances in muscle-*Sod2*^−/−^ mice are less attributable to glucose availability and the innervation of neuromuscular junctions.

## 4. Discussion

The proper regulation of energy production is necessary for normal skeletal muscle functioning. Glycolysis, which utilizes glucose and glycogen, is the main energy-producing pathway in fast muscles (Figure 1A). Glycogen is consumed during muscle activity and is re-synthesized from blood-borne glucose during muscle contractions [18,19]. GP-M decomposes glycogen and functions as a rate-limiting enzyme in muscle glycogenolysis. In the present study, we demonstrated that muscle-*Sod2*^−/−^ mice exhibited significant exercise intolerance due to reduced GP-M activity caused by mitochondrial redox imbalance. Indeed, glycogen significantly accumulated in the skeletal muscles of muscle-*Sod2*^−/−^ mice (Figure 1B,D), indicating a decrease in energy production through glycolysis. Notably, exercise intolerance in muscle-*Sod2*^−/−^ mice was caused by reduced GP-M activity, but not the reduced expression of GP-M (Figure 1B,C and Figure 2A). The DTT treatment of lysates from hPYGM-overexpressing cells reversed the decrease in GP-M activity caused by H_2_O_2_ treatment (Figure 3A), indicating that GP-M activity is reversibly regulated by the redox status. Regarding the relationship between superoxide and GP-M activity, it has been reported that hydrogen peroxide oxidized the cysteine bonds of GP-B and GP-M, and decreased their phosphorylase activity [20]. Since muscle-*Sod2*^−/−^ mice show functional abnormalities of skeletal muscle, especially in fast muscle, the physiological role of the oxidative inactivation of GP-M may be as a brake to mitigate muscle injury during excessive exercise. GP is also an allosteric enzyme, regulated by both phosphorylation and the binding of allosteric effectors, such as AMP [21,22]. The disulfide bond acts as a redox switch in the AMP-binding site and controls the activation of GP by AMP. In addition, various kinases are regulated by their redox state [23,24]. The effects of kinases upstream of GP-M and other glycogen metabolic enzymes in muscle-*Sod2*^−/−^ mice require further investigation. α-actinin-3, a fast muscle fiber protein, interacts with GP-M and regulates its activity [25]. Muscle-*Sod2*^−/−^ mice showed functional abnormalities in fast muscles, suggesting that α-actinin-3, cAMP/PKA, and multiple factors are involved in the combined reduction in GP-M activity. In the present study, antioxidants such as EUK-134 and PAPLAL improved exercise intolerance in muscle-*Sod2*^−/−^ mice by increasing GP-M activity (Figure 3D,E, Figure 4A,B and Figure S2). Antioxidants are expected to improve muscle function by targeting the GP-M activity.

Glycogen concentrations vary considerably across cell types, being highest in the liver and skeletal muscles and much lower in smooth muscle and other tissues [26,27,28,29]. In muscles, glycogen is heterogeneously distributed among three separate compartments: (I) subsarcolemmal glycogen just beneath the sarcolemma; (II) intermyofibrillar glycogen, located between the myofibrils, mainly at the level of the I-band close to the mitochondria and sarcoplasmic reticulum; and (III) intramyofibrillar glycogen in the myofibrils [30,31,32]. Heavy resistance exercise mediates the substantial utilization of glycogen from all three subcellular locations in type 2 fibers [33], suggesting that skeletal muscle glycogen content is associated with muscle function. In the sarcomeric and membrane organization of skeletal muscle, glycogen and mitochondria are distributed heterogeneously, with clustering found both in the subsarcolemmal space and intramyofibrillar space; glycogen is also found in the intermyofibrillar space [31]. The colocalization of glycogen and mitochondria indicates their close interaction with the muscle function. In fact, during exercise of varying loads, intermyofibrillar glycogen is utilized more preferentially, whereas disuse decreases glycogen in the intramyofibrils in association with the decreased mitochondrial content in the intermyofibrillar and subsarcolemmal compartments [30,33,34]. As glycogen granules were observed near mutant muscle mitochondria, increased superoxide or decreased ATP production from the mitochondria might impair glycogen metabolism around mutant mitochondria in the intermyofibrillar compartment.

Consistent with the mitochondrial dysfunction observed in muscle-*Sod2*^−/−^ mice reported in our previous study [3], muscle fibers from muscle-*Sod2*^−/−^ mice showed a marked decrease in mitochondrial respiration capacity (Figure S1B). Although muscle-*Sod2*^−/−^ mice exhibited significant exercise intolerance, the reduction in GP-M activity was only half that of controls (Figure 1C). These results suggest that the additive effects of reduced GP-M activity and mitochondrial dysfunction induce severe exercise intolerance in muscle-*Sod2*^−/−^ mice. Heart/muscle-specific and brain-specific *Sod2*^−/−^ mice show reduced mitochondrial Complex I and SDH activity [35,36]. The cGAS-STING signal is activated by sensing mtDNA leaking out due to damage to the inner mitochondrial membrane [37]. Damage to the mitochondrial inner membrane is thought to result in leakage of various TCA cycle metabolites. The mitochondrial proteins OMA1 and DELE1 sensed disruption of the inner mitochondrial membrane and, in response, activated the mitochondrial integrated stress response to increase the building blocks for protein synthesis [38]. These mitochondria-related factors may also be involved in regulating GP-M activity. Further research is needed on the interplay between mitochondria and glycogen in the muscle function.

In the present study, we were not able to perform comprehensive omics analyses, which limited the data from which conclusions could be drawn. In particular, we believe that if we could identify and structurally evaluate oxidatively inactivated GP-M using proteomics techniques, we could demonstrate a more detailed molecular mechanism of redox regulation of GP-M. In addition, qualitative and quantitative data on metabolites involved in glycogen metabolism by metabolomic analysis could more clearly elucidate the relationship between mitochondria and glycogen metabolism. Furthermore, rescue experiments using GP-M enzyme supplementation by *PYGM* gene transfer and analysis using human skeletal muscle are also expected to provide further insight into the results of this study.

## 5. Conclusions

**Figure 6:**
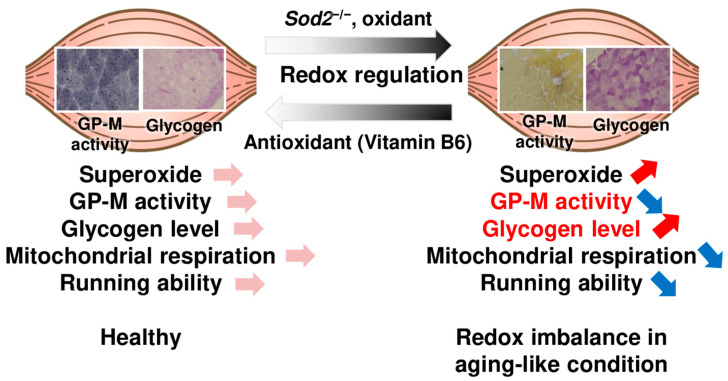
A schematic illustration of the summary. The mitochondrial redox imbalance in muscle-*Sod2*^−/−^ mice caused severe exercise intolerance associated with mitochondrial dysfunction, and glycogen accumulation via a loss of GP-M activity. The GP-M activity was suppressed by excess superoxide and was reversibly recovered by treatment with antioxidants or vitamin B6.

In conclusion, we demonstrated that the oxidative inactivation of GP-M is one of the causes of severe motor disturbances due to SOD2 deficiency (Figure 6). Further studies are required to determine the involvement of other glycogen metabolism-related enzymes. The induction of mitochondrial oxidative stress by various factors, such as lifestyle and aging, may cause muscle dysfunction due to abnormal glycogen metabolism, which is associated with decreased GP-M activity. The use of a redox modulator may be applicable to the enhancement of the muscle function, as a strategy targeting the regulation of glycogen metabolism.

## Figures and Tables

**Table 1:** PCR primers.

Gene Name	Forward	Reverse
*Pax7*	GACGACGAGGAAGGAGACAA	ACATCTGAGCCCTCATCCAG
*Myog*	CCTTGCTCAGCTCCCTCA	TGGGAGTTGCATTCACTGG
*Myod1*	GGCTACGACACCGCCTACTA	GTGGAGATGCGCTCCACTAT
*Mstn*	CTGTAACCTTCCCAGGACCA	TCTTTTGGGTGCGATAATCC
*Trim63*	GCCATCCTGGACGAGAAGAA	CAGCTGGCAGCCCTTGGA
*Fbxo32*	AGACCGGCTACTGTGGAAGAG	CCGTGCATGGATGGTCAGTG
*Gyg*	GGTGGCCTGACTGTTTCAAT	CAAATGGCAGTTTTGTG
*Gsk3* *β*	CCACATGCTCGGATTCAGGC	TGTCCACGGTCTCCAGCATTAGTAT
*Ugp2*	TGAGTTTGTCATGGAAGTCA	GATTTCCACCAGTCTCAGTT
*Gys1*	TCAGAGCAAAGCACGAATCCAG	CATAGCGGCCAGCGATAAAGA
*Gbe1*	ACTACCGAGTCGGGACAGCAA	GGTCCAGTCTCTGATGACCTCCATA
*Pygm*	CTTAGCCGGAGTGGAAAATGT	GTAATCTCTCGGAGTAGCCACA
*Agl*	ACTGTGGCACGTGGATGGATAA	CCCACGATTTCCACAGCAGA
*Hk1*	AACCTCAAAGTGACGGTGGGC	AAGGACACGTCACATTTCGGAGC
*Pfkfb3*	AGAACTTCCACTCTCCCACCCAAA	AGGGTAGTGCCCATTGTTGAAGGA
*Slc2a*	CAACTGGACCTGTAACTTCATCGT	ACGGCAAATAGAAGGAAGACGTA
*H2a*	ACGAGGAGCTCAACAAGCTG	TATGGTGGCTCTCGGTCTTC

*Pax7*; paired box 7, *Myog*; myogenin, *Myod*; myogenic differentiation 1, *Mstn*; myostatin, *Trim63*; tripartite motif-containing 63, *Fbxo32*; atrogin-1, *Gyg*; glycogenin, *Gsk3β*; glycogen synthase kinase 3 beta; *Ugp2*; UDP-glucose pyrophosphorylase 2,*Gys1*; glycogen synthase 1, muscle, *Gbe1*; glucan (1,4-alpha-), branching enzyme 1, *Pygm*; muscle glycogen phosphorylase; *Agl*; amylo-1,6-glucosidase, 4-alpha-glucanotransferase; *Hk1*; hexokinase 1, *Pfkfb3*; 6-phosphofructo-2-kinase/fructose-2,6-biphosphatase, *Slc2a*; Glucose transporter 4, *H2a*; histone H2A.

## Data Availability

Data are contained within the article.

## Supplementary Materials

The following supporting information can be downloaded at: https://www.mdpi.com/article/10.3390/antiox13111421/s1, Figure S1: Mitochondrial superoxide generation and respiration in muscle-*Sod2*^−/−^ mice; Figure S2: Treadmill task performance of EUK-134, an SOD mimetic, on muscle-*Sod2*^−/−^ mice; Figure S3: Full blot images of Western blotting.
