# Supplementary material for: Mitochondrial Redox Status Regulates Glycogen Metabolism via Glycogen Phosphorylase Activity
Source: Antioxidants (Basel). 2024 Nov 20;13(11):1421. doi: 10.3390/antiox13111421 (PMC11590902; doi:10.3390/antiox13111421)
Supplement: Supplementary file 1 [file antioxidants-13-01421-s001.zip › antioxidants-3252780-supplementary.pdf]

Supplemental materials and methods

Dihydroethidium (DHE) staining

Muscle superoxide levels were analyzed by DHE staining (Nacalai Tesque, Inc., Kyoto, Japan). Briefly, frozen sections (6 μm) were dried and incubated in 10 μM DHE solution at room temperature for 30 min.

Measurement of oxygen consumption rate (OCR)

The OCR of the muscle fibers isolated from the gastrocnemius was measured using an O2k-FluoRespirometer (Berthold Japan, Tokyo, Japan) according to the manufacturer's instructions.

EUK-134 treatment in vivo

EUK-134 (Axon MEDCHEM, Groningen, the Netherlands) dissolved in PBS (0.25 mg/ml was intraperitoneally injected into muscle-*Sod2*$^{-/-}$ mice at a dose of 35 mg/kg body weight. During the treadmill task, all mice were habituated to running for several days until their endurance time stabilized. The drug was administered 24 h before the experiment. The treadmill task was performed 24 h after injection.

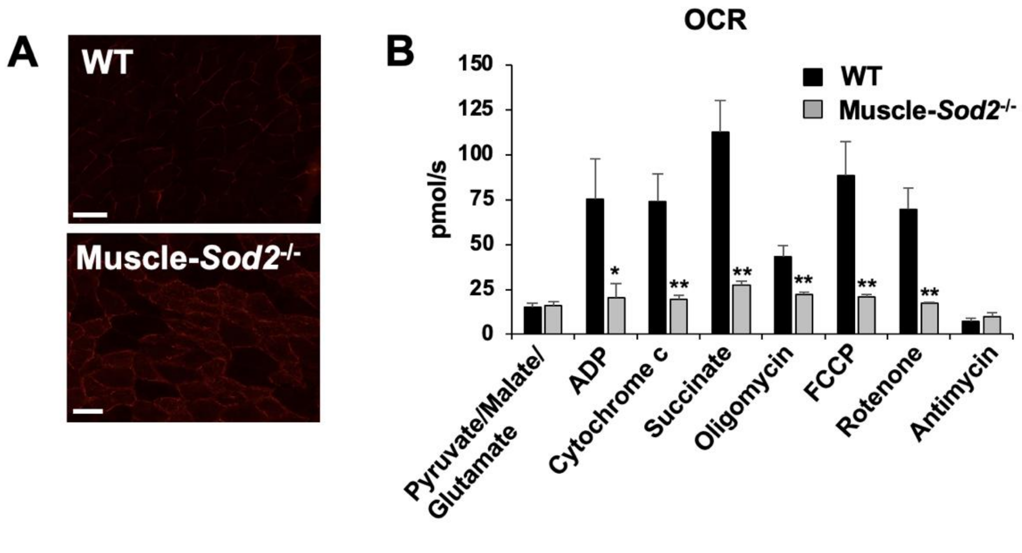

Figure S1. Mitochondrial superoxide generation and respiration in muscle-*Sod2*$^{-/-}$ mice. (A) Mitochondrial superoxide staining by DHE (dihydroethidium) of the gastrocnemius muscle of WT and muscle-*Sod2*$^{-/-}$ male mice at 5-6 months of age. Scale bars represent 50 μm. (B) Oxygen consumption rate (OCR) of muscle fiber isolated from the gastrocnemius of WT and muscle-*Sod2*$^{-/-}$ male mice (n = 3). Data are shown as the mean ± SD; $^{*}p < 0.05$, $^{**}p < 0.01$.

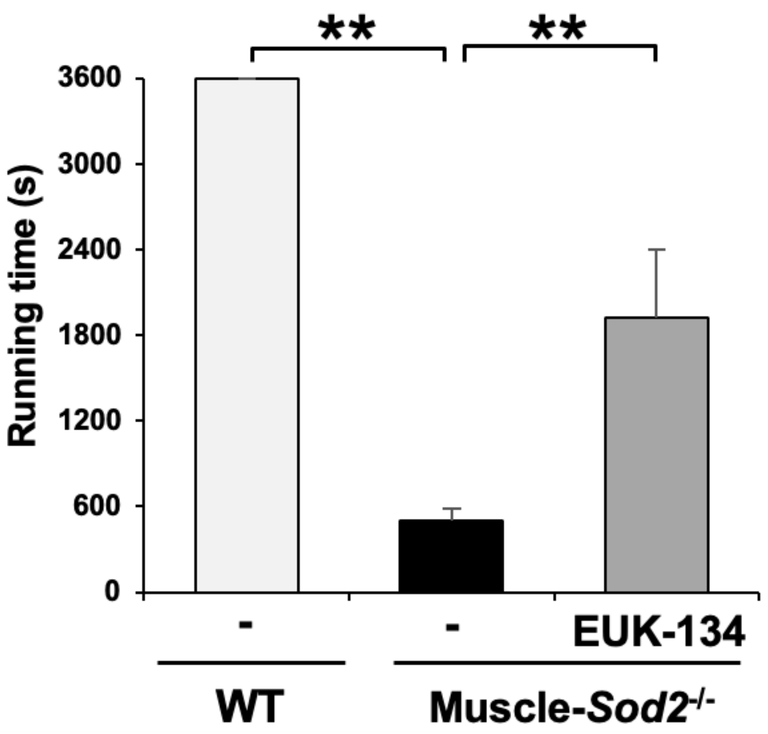

Figure S2. Treadmill task performance of EUK-134, an SOD mimetic, on muscle-*Sod2*$^{-/-}$ mice. Treadmill task performance of WT and muscle-*Sod2*$^{-/-}$ mice with or without administration of the antioxidant EUK-134 at 5 months of age (n = 5). Data are shown as the mean ± SD; $^{**}p < 0.01$.

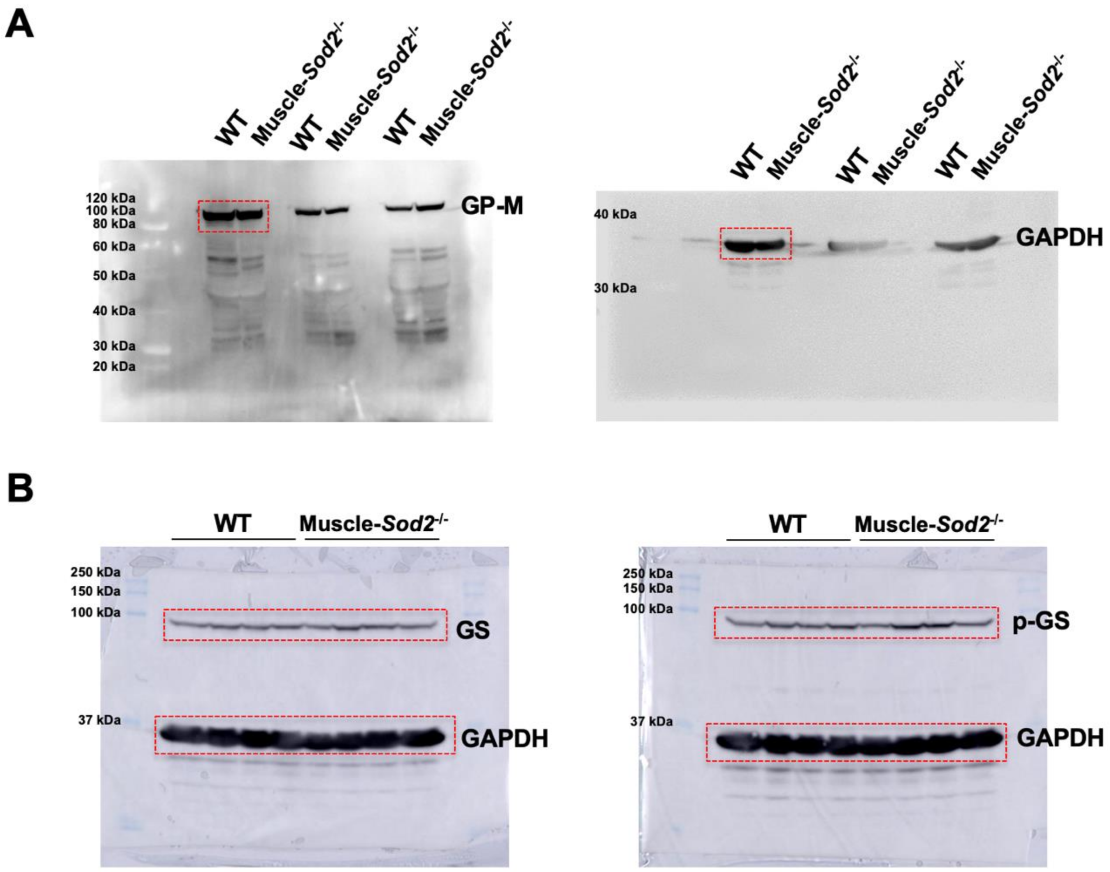

Figure S3. Full blot images of western blotting. Full blot images of Figure 2A (A) and Figure 2C (B).
